# Development of immersive learning framework (ILF) in achieving the goals of higher education: measuring the impact using a pre–post design

**DOI:** 10.1038/s41598-023-45035-0

**Published:** 2023-10-17

**Authors:** Saju Madavanakadu Devassy, Lorane Scaria, Jed Metzger, Kiran Thampi, Jitto Jose, Binoy Joseph

**Affiliations:** 1 Department of Social Work, Rajagiri College of Social Sciences (Autonomous), Rajagiri P.O, Kalamassery, Kochi, Kerala 683 104 India; 2 Rajagiri International Centre for Consortium Research in Social Care, Rajagiri College of Social Sciences (Autonomous), Kochi, Kerala India; 3 Social Work Department, Nazareth College, 4245 East Avenue, Rochester, NY 14618 USA; 4 Office of International Relations, Rajagiri College of Social Sciences (Autonomous), Kochi, India; 5 Department of Statistics, Rajagiri College of Social Sciences (Autonomous), Kochi, India; 6 Rajagiri Business School, Rajagiri Valley, Kochi, Kerala India

**Keywords:** Psychology, Risk factors

## Abstract

Emerging technological tools like Artificial Intelligence-based Chatbots, digital educational alternatives and market-driven educational systems pose a challenge to the fundamental aim of the higher education system; comprehensive education for well-being. Therefore, this research aims to devise and evaluate strategies to impart new-age competencies to innovate socially and morally appropriate solutions in a modern competitive innovative society. The 8-month-long immersive learning framework (ILF), was designed based on the volatility, uncertainty, complexity, and ambiguity (VUCA) paradigm. The framework was evaluated with 133 newly joined postgraduate students doing their science or arts programmes from a higher education institution in Kerala, India. The outcome variables included well-being, depressive symptoms, personality patterns, and sub-domains of philosophy of human nature. The follow-up scores showed a significant improvement in well-being (Mean difference: 1.15, p = 0.005), trustworthiness (Mean difference: 14.74, p = 0.000), strength of will (Mean difference: 10.11, p = 0.000), altruism (Mean difference: 12.85, p = 0.000), and independence (Mean difference: 11.93, p = 0.000). Depression scores did not improve significantly. However, the intervention shielded them from the adjustment issues that often accompany any transition. The ILF framework can help students develop their personal and professional selves if it is implemented collaboratively in a reflective setting. It can also instil moral rectitude and a prosocial mindset.

## Introduction

Universities historically played a critical role in social, political, cultural, economic, and scientific breakthroughs to raise people’s living standards and well-being^1,2^. The market-driven educational system poses a challenge to the fundamental aim of education. In order to make students active learners with a social and moral conscience, India’s new National Education Policy 2020 emphasises the development of holistic and multidisciplinary learning with a focus on competency enhancement and experiential learning^3^. This movement is very well aligned with the Indian Vedic academic tradition—Gurukul (an ancient Indian education system where the students resided at the teacher’s (guru) house and had experiential learning. The emotional bond between teacher and student was considered critical in achieving the goal of acquiring knowledge for well-being)—which demonstrated a positive and peaceful reflective immersive environment to develop students into men and women of character with healthy minds, refined hearts, and disciplined spirits^4^. Through various exposures, they learn the ideals of universal brotherhood/sisterhood, humanity, love, discipline, truthfulness and mindfulness. The ideology of balance that Gurukul upheld focused on informed decisions in life, interpersonal relationships, and work-life to be happy and stress-free, which is a prerequisite for addressing the challenges of a modern competitive innovative society^5,6^.

The increasing popularity of technological tools like Artificial Intelligence powered Chatbots, and other digital alternatives pose challenges to conventional teaching techniques, raise ethical concerns and even question the purpose of education itself^7^. Honesty, openness to learning, and a sincere interest in comprehending and meeting the needs of others are the survival capabilities we need in this scenario. Therefore, as in the Gurukul tradition, the faculty members are the key to revising the learning objectives and programs aligned with ethical, critical and creative systems thinking, to continuously innovate survival strategies^8^.

The immersive learning framework (ILF) is an experiential learning pedagogy to develop new age competencies using a praxis (know-act-reflect) paradigm. This is particularly crucial in the current scenario where youth are increasingly lacking employability skills^9^ and facing a multitude of issues in personal and professional domains^10^. ILF trains the students’ mental and logical capabilities through self-discovery, analysis of social and cultural contexts, navigating contextual resources, and collectively innovating situated activities, that help in acquiring the skill of informed decision-making.

The VUCA (Volatility, Uncertainty, Complexity, and Ambiguity) framework^11,12^, was used to design the action component because the COVID-19 stuck world demanded people to be adaptable and responsive to unforeseen circumstances as a survival strategy. Personal, interpersonal, and social issues and concerns were the primary targets of the intervention components. This targeted intervention framework was cocreated in consultation with the users. Volatility compels students to steer their purpose and meaning in line with the vision of higher education. Instilling an openness to learn from faculty, experienced elders, experts, local leaders, and the community helps the students to make informed decisions during volatility. Uncertainty is addressed through an in-depth insight into their capabilities and equipping students to convert any uncertainty into their advantage. It is done through a process of frequent communication, and feedback to the group members about the latest developments to adapt to the current needs. Complexity deals with creating opportunities for experiences, requiring the courage to take calculated risks and daring decisions, especially when they find gaps in already available information and specific requirements of unpredictable ground realities. The team responds to ambiguity with a range of partialized plans—partialize the major goals into smaller ones and devise strategies to address each separately to facilitate easier course alterations. Table 1 describes the intervention framework.

**Table 1:** Intervention framework.

Context	Purpose	Content	Structure
Volatility	A clear vision statement	Steer the goals in line with the vision	Instilling the mission, vision and values to the students to set flexible goals in times of volatility
Uncertainty	Adaptability to any situations	The continual updating and gathering of information to make decisions	Insight into their capabilities and equipping students to convert any uncertainty into their advantage
Complexity	Provide clarity	Consolidate teamwork through communication to deal with contradictory information and perspectives	Consolidate the information to make bold, future-oriented decisions to ensure success
Ambiguity	Promote agility	Consolidate information and its contradictory interpretations and get the team to act	Keep the people’s felt needs at the centre of their decisions

The study’s premise is that if an institution focuses on a mission, vision, and value system based on citizenship values, humanist qualities like integrity, openness to new experiences, and genuine concern for others (altruism) could be transmitted through a systematic integrated process. The current study describes the intervention model and tests its effectiveness in developing collectivist values and employability skills to ensure personal and professional well-being.

## Methodology

### Study design

A single-arm pre-post design was used to evaluate the feasibility of an immersive learning framework developed over the course of 20 years. The study was conducted between July 2022 and March 2023 with the postgraduate students of a government-funded autonomous college.

### Study setting and sample

The study was carried out in a catholic religious congregation-founded, secularly run, higher education institution in Ernakulam, Kerala, South India, which has always upheld a distinctive social sensitivity ethos. The authors used a broad inclusion criterion for the study. The participants included all 133 postgraduate students who had at least 75% attendance in 75% of activities and were enrolled in the first year of the 2-year Social Work, Statistics, Master of Computer Applications (MCA), and Master of Data Sciences (MSc Data Sciences) programmes at the institute for the academic years 2022–2024.

### Measurements

Age, gender, religion, place of residence, life situation, and programme opted were all included in the demographic information. The students were broadly divided into two groups: the ‘Science’ students, who were enrolled in MCA, MSc Data Sciences and Statistics programs, and the ‘Arts’ students, who were enrolled in Social Work programs.

### Primary outcome measures

#### Well-being

The respondents’ well-being was measured using the WHO Wellbeing Index (WHO-5). The tool consists of five statements measured on a six-point rating scale ranging from all of the time (5) and at no time (0) with a total score ranging between 0 and 25^13^. WHO-5 is a reliable measurement validated in India with a Cronbach alpha score of 0.794^14^.

#### Depression symptoms

The depression level of respondents is measured using the patient health questionnaire (PHQ-9). PHQ 9 is a set of nine questions that measure the level of depressive symptoms ranging between “0” (not at all) to “3” (nearly every day). The total scores range from 0 to 27, with 0 representing the least severe depressive symptoms and 27 signifying the most severe. Internal consistency reliability (Cronbach’s alpha 0.89; 95% confidence interval (CI) 0.86–0.95) and interrater reliability (interclass correlation coefficient, 0.94; 95% CI 0.86–0.95) were both good. This assessment tool was validated in India^15^.

### Secondary outcome measures

#### Personality

The personality attributes of the respondents were measured using the Big Five Inventory (BFI-44). The BFI^16^ is a reliable and valid instrument that measures five personality traits, extraversion—energetic, sociable, enthusiastic, and outgoing (vs introversion), agreeableness—sympathetic, forgiving, and warm (vs antagonism), conscientiousness—organized, efficient, and thorough (vs lack of direction), neuroticism—tense, moody, irritable and not contented (vs emotional stability) and openness—curious, imaginative, artistic, excitable and unconventional (vs closedness to experience). The tool is a set of 44 questions assessed on a five-point Likert scale ranging from ‘disagree strongly’ (1) to ‘agree strongly’ (5). Extraversion is measured by a set of eight questions (1, 6R, 11, 16, 21R, 26, 31R, 36; R denotes negative scoring), agreeableness through (2R, 7, 12R, 17, 22, 27R, 32, 37R, 42), conscientiousness by a set of nine questions (3, 8R, 13, 18R, 23R, 28, 33, 38, 43R), neuroticism by eight questions (4, 9R, 14, 19, 24R, 29, 34R, 39) and openness by a set of 10 questions (5, 10, 15, 20, 25, 30, 35R, 40, 41R, 44). A higher score indicated extraversion over introversion, agreeableness over antagonism, conscientiousness over lack of direction, neuroticism over emotional stability, and openness over closedness to experience. The BFI has been shown to have good psychometric properties with a reliability coefficient of 0.83^17^.

#### Philosophy of human nature

Philosophies of human nature scale (PHN) was used to measure philosophical nature in six domains, namely, trustworthiness (vs untrustworthiness), the strength of will and rationality (vs lack of willpower and irrationality), altruism (vs selfishness), independence (conformity to group pressures), complexity (vs simplicity), and variability (vs similarity). The tool consists of 84 questions, 14 questions for each domain, measured on a six-point rating scale ranging from “strongly disagree” (− 3) to “strongly agree” (3). Scores for each domain range between − 42 and 42, where a positive value indicates trustworthiness, the strength of will, altruism, independence, complexity, and variability, while a negative value denotes untrustworthiness, lack of willpower, selfishness, conformity, simplicity, and similarity. The tool was reliable (Cronbach’s alpha = 0.64) and valid^18^.

### Intervention model—the immersive learning framework

The 1-year intervention programme was designed to offer experiential learning opportunities based on various culturally relevant behaviour modification theories. This immersive learning framework was underpinned by Talcott Parsons’ functionalist theory^19^ which advocated the collective orientation against divisive forces endangering the team process. This framework was aligned with the Eastern collectivist ideology, which strongly emphasised preferential options for the welfare of others as a prerequisite for individual well-being^20^.

The ILF framework consists of a series of carefully designed activities including (a) faculty induction to the framework; (b) know your neighbourhood, (c) Rural sensitization camp; (d) Vanavasam and (e) Kalipso and (f) organizing or participating in the observance, of at least two important days, such as women’s day, disability day, red ribbon day to name a few (Table 2). Vanavasam—literally means withdrawing to the forest, is conceived as an intense personality development programme focusing on self-introspection, wellness, and openness to self and others. The program comprises various mindfulness-focused exercises and self-awareness programs based on the Johari Window framework. The program ends with a contract the students make with themselves on how to improve themselves in the future, which makes them committed to lifelong learning. Kalipso is a 3-day leadership game-based programme designed to foster unconventional lateral thinking and teamwork to innovate solutions, collaborative implementation of designed solutions, and reflection on the underlying values.

**Table 2:** Detail description of activities and indicators of intervention.

VUCA	Goals	Activities	Indicators
Volatility	Conceptual learning	1. Faculty preparedness through academic planning 2. Student competency gap assessment 3. Induction program (a) Management perspective (b) Faculty perspectives	1. Faculty stay outside the college to reflect on the vision and mission and devise strategies to implement it—5 days × 8 h = 40 h 2. Pre-assessment for students—1 h 3. One week-long induction program (a) Management deliberates on vision, mission, and future direction—1 day × 6 h = 6 h (b) Faculty inducted to immersive learning experience—3 days × 6 h = 18 h
Uncertainty	Experiential engagements	(a) Know your neighbourhood (b) Vanavasam—(It is a self-assessment and guided reflection process based on the Johari window, designed to encourage self-awareness and self-discovery in a reflective learning and practice context, ultimately result in continuous improvement of their performance) (c) Kalipso—a three-day leadership game-based programme	(a) Visiting, interacting and reporting on at least 20 government and significant organizations in the neighbourhood—6 days × 6 h (b) Staying in an accommodation facility in a forest area for self-exploration in an interpersonal context—2 days × 9 h = 18 h (c) To foster lateral thinking, teamwork, collaborative implementation, and value reflection—3 days × 9 h = 27 h
Complexity	Executive modelling and civic engagements	(a) Orientation (b) Student classification (c) Tailormade mentoring based on grouping (d) Project identification in consultation with the community leaders, people and other stakeholders	(a) Orientation on the rural sensitivity; vision, context, purpose, structure, content, and process—7 days × 3 h = 21 h (b) Student classifications and grouping based on the transtheoretical model of behaviour change^47^—1 h (c) Individual mentoring sessions to align students with mission—1 h (d) Need assessment, prioritizing, setting goals and objectives, devising activities, strategizing the implementation, and evaluation—10 h
Ambiguity	Professional competency	(a) Pre-camp (b) Camping days and daily camp evaluation (c) Post-camp evaluation and assessment (d) Engagement in any of the farming activities or community survey (e) Post assessment	(a) Practical preparations (b) Implementation—deployment of students, allocation of project, home visits, cultural evening, reflection—7 days × 10 h (c) Evaluation and reporting submission of the reports—1 day × 6 h (d) Farming activities—field preparation, planting or harvesting or socioeconomic and health survey—2 days × 8 h (e) Post-assessment after 1 year

### Statistical analysis

Statistical analysis was performed using SPSS (IBM Version 25, New York, NY, USA) and the Stata (StataCorp LLC Version 15, Lakeway Drive, TX, USA) program. Descriptive statistics were used to identify the mean scores and frequencies of the major variables. Furthermore, chi-square tests were performed to explore the characteristics of the sample by the subgroups of Arts or Science. Results from the outcome measurements were summarized, and the differences between the baseline and follow-up measurements were determined using a paired t-test. The effect size of each test was calculated using Cohen’s *d.* Outcome scores pre- and post-intervention were calculated, and participants who had an increase in the score from baseline were labelled as “participants with increased outcome scores” (Yes and No) for well-being, trustworthiness, the strength of will, altruism and independence. Multivariate repeated measures ANOVA was used to determine the significance of the combined effect of the primary and secondary outcome variables. Logistic regression was performed using “Participants with increased well-being scores from baseline” as the dependent variable to assess the factors associated with the increase.

### Ethical considerations

Before involving the students in the current study, their written informed consent was obtained. Confidentiality and anonymity were maintained throughout the process. The study was performed in accordance with the relevant guidelines and regulations of the Declaration of Helsinki. Ethics committee approval for the exploratory trial was obtained from the Institutional Review Board (IRB) of Rajagiri College of Social Sciences.

## Results

The mean age of the participants was 21.9. Students belonged to the arts (42.1%) and science (57.9) streams. The majority of students were females—78% from the arts stream and 61% from the science stream were females. The participants represented the cross-section of the state in terms of religious affiliation and place of residence. The majority of them belonged to the average income category (75%) (Table 3).

**Table 3:** Demographic characteristics.

Variables	Total (n = 133)	Division (N = 133)		Statistics, p value
		Arts (N = 56)	Science (N = 77)	
Age (mean, S.D)	21.87 (3.06)	22.57 (4.50)	21.37 (0.98)	T = 2.25, p = 0.02
Gender				chi2 = 4.61, p = 0.032
Female	91 (68.42%)	44 (78.57%)	47 (61.04%)	
Male	42 (31.58%)	12 (21.43%)	30 (38.96%)	
Religion				chi2 = 7.70 p = 0.021
Hindu	58 (43.61%)	23 (41.07%)	35 (45.45%)	
Muslim	12 (9.02%)	1 (1.79%)	11 (14.29%)	
Christian	63 (47.37%)	32 (57.14%)	31 (40.26%)	
Place of birth/residence				chi2 = 1.95 p = 0.376
Town	48 (36.09%)	24 (42.86%)	24 (31.17%)	
Village	44 (33.08%)	17 (30.36%)	27 (35.06%)	
City	41 (30.83%)	15 (26.79%)	26 (33.77%)	
Income category*				
Average income group	101 (75.94%)	43 (76.79%)	58 (75.32%)	chi2 = 0.03, p = 0.846
High-income group	32 (24.06%)	13 (23.21%)	19 (24.68%)	

*According to monthly income, participants were divided into quartiles; the first three quartiles were labelled as the average income group, and the last quartile was labelled as the high-income group.

Mean scores for extraversion, agreeableness, conscientiousness, neuroticism, and openness were 26.9, 37.5, 31.9, 23.9, and 35.1, respectively. Mean scores for the subdomains of philosophical natures were − 2.4 (S.D = 8.3), 1.9 (S.D = 7.6), 3.8 (S.D = 8.2), 4.7 (S.D = 6.2), respectively for trustworthiness, the strength of will, altruism, and independence (Table 4).

**Table 4:** Mean scores of personality subdomains and major outcome variables at baseline.

	Total (n = 133)	Range
Personality type		
Extraversion score	26.89 (4.99)	13 to 38
Agreeableness score	37.51 (4.35)	25 to 45
Conscientiousness score	31.87 (4.91)	17 to 45
Neuroticism score	23. 87 (5.98)	9 to 40
Openness score	35.11 (3.99)	26 to 44
Wellbeing scores	15.3 (4.9)	5 to 25
Depression scores	7.5 (4.9)	0 to 21
Philosophy of human nature		
Trustworthiness score	− 2.44 (8.30)	− 28 to 17
Strength of will score	1.91 (7.55)	− 19 to 28
Altruism score	3.83 (8.17)	− 17 to 52
Independence score	4.67 (6.24)	− 15 to 20

A multivariate repeated measures ANOVA showed a significant change from pre to post on a weighted linear combination of the six outcome variables (Pillai’s trace = 0.729, Exact F = 56.9, p < 0.000). Univariate analysis of each outcome variable based on effect by time analysis yielded the following results (Table 5). The effect by time analysis was significant for all the variables except depression. The results indicate that the intervention significantly improved well-being, and philosophical considerations of trustworthiness, strength of will, altruism, and independence among the students.

**Table 5:** Univariate analysis of the major outcome variables (effect by time analysis).

	Mean square	F	p value
Wellbeing baseline–wellbeing post-intervention	89.15	6.67	0.011
Depression baseline–depression post intervention	25.27	1.90	0.170
Trustworthiness baseline–trustworthiness post-intervention	14,456.84	300.16	0.000
Strength of will baseline–strength of will post-intervention	6800.84	75.05	0.000
Altruism baseline–altruism post-intervention	10,980.01	148.76	0.000
Independence baseline–independence post-intervention	9468.31	176.94	0.000

Table 6 indicates the pre-post results of the primary and secondary outcome measures of the study. There was a statistically significant improvement in the average well-being scores from baseline to follow-up measurement (Mean difference: 1.15, p = 0.005). Further, mean scores of trustworthiness (Mean difference: 14.74, p = 0.000), the strength of will (Mean difference: 10.11, p = 0.000), altruism (Mean difference: 12.85, p = 0.000), independence (Mean difference: 11.93, p = 0.000) increased from baseline to follow up. All the associations were statistically significant. A high effect size difference was observed for all philosophical considerations. Out of the total, 54% showed an increase in well-being scores, which was the major primary outcome of the current study.

**Table 6:** Pre- and post-test scale scores and their comparisons of major outcome variables.

Variables/outcomes	Baseline Mean (SD)	Post intervention Mean (SD)	Mean of difference (post vs baseline)			Cohen’s d effect size	Participants achieved better outcomes n (%)
			Mean	95% CI	*p*		
Wellbeing	15.3 (4.9)	16.5 (4)	− 1.2	(− 2.0, − 0.3)	0.005	− 0.26	72 (54.1%)
Trustworthiness	− 2.4 (8.3)	12.3 (7.8)	− 14.7	(− 16.4, − 13.1)	< 0.001	− 1.82	124 (93.2%)
Strength of will	1.9 (7.5)	12.0 (9.7)	− 10.1	(− 12.4, − 7.8)	< 0.001	− 1.16	103 (77.4%)
Altruism	3.8 (8.2)	16.7 (9.1)	− 12.9	(− 14.9, − 10.8)	< 0.001	− 1.49	114 (85.7%)
Independence	4.7 (6.2)	16.6 (8.6)	− 11.9	(− 13.7, − 10.1)	< 0.001	− 1.58	114 (85.7%)

Binary logistic regression was performed with the participants who had an improvement in wellbeing scores as the outcome variable and other demographic and personality variables as the independent variables to identify the significant factors associated with an increase in wellbeing. Results (Table 7), revealed that no factor—age, gender, academic stream, income category, or personality was statistically significantly associated with the increase in well-being. The intervention improved well-being among 72 participants irrespective of their demographic or personality characteristics.

**Table 7:** Factors associated with an increase in well-being among participants (binary logistic regression).

Factor	Crude odds ratio	95% confidence interval	p value
Age	1.04	(0.91–1.18)	0.560
Gender			
Female	Ref		
Male	1.37	(0.65–2.88)	0.398
Division			
Arts	Ref		
Science	1.94	(0.96–3.90)	0.062
Income category			
Average income group	Ref		
High-income group	1.57	(0.69–3.54)	0.278
Personality			
Extroversion score	0.97	(0.90–1.04)	0.434
Agreeableness score	0.96	(0.89–1.04)	0.415
Conscientiousness score	1.02	(0.95–1.09)	0.578
Neuroticism score	1.01	(0.94–1.06)	0.879
Openness score	0.96	(0.88–1.05)	0.404

## Discussion

The immersive learning framework was developed against higher education’s transition from an all-encompassing, wellbeing-focused communitarian value-oriented system to a profit-oriented market-driven individualistic model. In order to regain the traditional higher education orientation, the current framework provides safe and gradual exposures to various prosocial, self-exploratory experiences that enable a person to analyse social reality objectively, and creatively engage in activities to develop the skills and competencies.

The covid pandemic, technological developments like Chatbots powered by artificial intelligence and digitised learning^21^, socioeconomic challenges like economic stratification and disparities^22^, and geopolitical issues^23^ all pose newer challenges to achieving the goals of higher education and gaining employability skills. Participants in the model go through demanding, unstructured environments with opportunities for anticipatory guidance, which has been proven effective in preparing the students for future challenges in both their personal and professional lives^24^. Conceptual learning, experiential engagements, executive modelling, and civic participation were all part of the process of professional and personal success and well-being.

In response to the emerging global agenda of promoting overall well-being in higher education^25^, the current innovation helps in acquiring skills of informed decision-making and improving self-efficacy which is crucial for developing employability skills^26^. The Vanavasam, Kalipso and Rural sensitization camp was designed for holistic development and to impart Gurukul values of universal brotherhood/sisterhood, discipline, truthfulness, mindfulness about nature and society, openness to learning, and sincere interest in the well-being of others.

The programs in the model are designed in three levels, self-reflection-oriented individual programs, focused group activities and social sensitivity-based community engagements^27–29^. These activities are intertwined and cumulatively provide immersive learning experiences to students. Vanavasam, modelled after the Johari Window framework, fosters introspection, self-reflection, and self-realisation^30,31^. Mirroring the blind spots through feedback in a controlled, supportive interpersonal environment creates empathy and trust in relationships. This lays the foundation for their lifelong journey of self-discovery that enables them to survive potentially volatile situations.

Kalipso encourages lateral thinking to analyse the situation, innovate and implement solutions, and reflect on them based on humanistic and collectivistic ideals and ethics. The transtheoretical model of behaviour modification was used to design the rural sensitization camp, to enhance social sensitivity, group participation, and self-reflection. The package was designed based on the Theory of Change model of developing complex interventions^32^. Stages consisted of Identifying socially relevant infrastructural, environmental and developmental needs, prioritising, setting goals and objectives, devising activities and indicators, strategizing the implementation, and evaluating outputs and outcomes. Home visits were pivotal in understanding human nature and appreciating the rural collectivist social contexts and their values.

Furthermore, the farming engagements or community surveys expose the students to the reality of human life to help them understand how to contextualise social concerns within the framework of sustainable development goals (SDG). The ‘Know your neighbourhood’ assignment was another strategy to identify the contextual resources, both governmental and non-governmental, to plan situated activities for the community population. Important days are observed as a means of fostering prosocial mindsets and behaviors. Examples include observing Disability Day to foster an inclusive and empathetic mindset, Women’s Day to protect women’s dignity and value their contributions, and Red Ribbon Day to learn about and support people affected by HIV.

Post-assessment data showed that the intervention successfully enhanced most human nature subdomains, including trustworthiness, the strength of will, independence, altruism, and well-being of the participants from baseline to follow-up. In contrast to the female participants in our study, the male participants exhibited greater improvement across all areas of the philosophy of human nature. Though the reduction in depressive symptoms was not significant in either gender, there was a trend towards improvement in the post-assessment scores in both groups. As a comparison to students from the science stream, those who chose social science programmes had higher baseline levels of extroversion, consciousness, and openness to experiences. It might be because of the differences in the viewpoints of students studying science and social sciences. The science students’ scores on the subscales of the philosophy of human nature, however, were significantly higher after the intervention compared to the Arts students’ scores. The reason could be that science students are exposed to fewer prosocial events than students in the Arts.

Many educators and educational institutions have experimented with experiential-learner-centred models due to their widespread acceptance^33^. They use various experiential learning methods such as service learning^34,35^, problem-based learning^36,37^, action learning^38,39^ adventure education^40,41^ and simulation and gaming^42,43^. ILF is another experiential learning process, where the students transform themselves through learn, act and reflect paradigm, which makes them lifelong receivers and creators of knowledge, thereby becoming successful graduates and promising citizens^44^. However, the effectiveness of ILF experiential learning model lies in the unique relationship between the teacher and the learner.

Most students who had gone through immersive learning framework (ILF) showed significant improvement in most areas of the philosophy of human nature, particularly in the areas of altruism and prosocial attitude, which was the main goal of this intervention. Hence, regardless of gender, socioeconomic status, or academic programme selection, the ILF intervention effectively enhances the primary outcome variables. This aligns with earlier research demonstrating that prosocial behaviours promote psychosocial adjustment^45^ lessen the likelihood of developing depression, and enhance general well-being and mental health^46^.

### Limitations of the study

The current immersive pedagogical framework to improve personal and professional well-being is the first of its kind to our awareness. The model caters to multiple dimensions of students’ academic life to develop them into socially responsible citizens of society. However, the study has its limitations as well. A randomised control trial could have prevented potential biases arising from a single-arm pre-post design. As the control group was not feasible in the given situation, the authors forego a true experimental design. Also, a comparably smaller sample size may have an impact on how generalizable the findings of the study are. Future research, thus, should aim at a cluster randomised trial with a larger sample in multiple centres before scaling up the intervention to newer areas.

## Conclusion

The all-encompassing wellbeing-focused, humanistic value-oriented Immersive Learning framework educates future professionals to develop competencies to design solutions to address the VUCA reality by gradual exposure to varied prosocial, self-exploratory experiences. The experiential know-act-reflect approach offers possibilities for teachers and students to forge a unique relationship in a self-reflective and introspective context. Therefore, the ILF model if contextually designed and implemented has the capacity to impart new-age competencies to innovate socially and morally appropriate strategies. Additionally, the objectives of competency-based holistic learning, as envisioned by India's new National Education Policy 2020, are best achieved through this experiential approach.

## Data Availability

The data are available on request from the corresponding author upon reasonable request.
